# The trade-off of availability and growth inhibition through copper for the production of copper-dependent enzymes by *Pichia pastoris*

**DOI:** 10.1186/s12896-016-0251-3

**Published:** 2016-02-20

**Authors:** Palanisamy Athiyaman Balakumaran, Jan Förster, Martin Zimmermann, Jayachandran Charumathi, Andreas Schmitz, Eik Czarnotta, Mathias Lehnen, Suresh Sudarsan, Birgitta E. Ebert, Lars Mathias Blank, Sankaranarayanan Meenakshisundaram

**Affiliations:** Centre for Biotechnology, Anna University, Sardar Patel Road, Guindy, Chennai 600025 India; iAMB - Institute of Applied Microbiology, ABBt – Aachen Biology and Biotechnology, RWTH Aachen University, Worringerweg 1, 52074 Aachen, Germany

**Keywords:** *Pichia pastoris*, Copper, Laccase

## Abstract

**Background:**

Copper is an essential chemical element for life as it is a part of prosthetic groups of enzymes including super oxide dismutase and cytochrome c oxidase; however, it is also toxic at high concentrations. Here, we present the trade-off of copper availability and growth inhibition of a common host used for copper-dependent protein production, *Pichia pastoris*.

**Results:**

At copper concentrations ranging from 0.1 mM (6.35 mg/L) to 2 mM (127 mg/L), growth rates of 0.25 h^−1^ to 0.16 h^−1^ were observed with copper uptake of as high as 20 mg_copper_/g_CDW_. The intracellular copper content was estimated by subtracting the copper adsorbed on the cell wall from the total copper concentration in the biomass. Higher copper concentrations led to stronger cell growth retardation and, at 10 mM (635 mg/L) and above, to growth inhibition. To test the determined copper concentration range for optimal recombinant protein production, a laccase gene from *Aspergillus clavatus* [EMBL: EAW07265.1] was cloned under the control of the constitutive glyceraldehyde-3-phosphate (GAP) dehydrogenase promoter for expression in *P. pastoris*. Notably, in the presence of copper, laccase expression improved the specific growth rate of *P. pastoris*. Although copper concentrations of 0.1 mM and 0.2 mM augmented laccase expression 4 times up to 3 U/mL compared to the control (0.75 U/mL), while higher copper concentrations resulted in reduced laccase production. An intracellular copper content between 1 and 2 mg_copper_/g_CDW_ was sufficient for increased laccase activity. The physiology of the yeast could be excluded as a reason for the stop of laccase production at moderate copper concentrations as no flux redistribution could be observed by ^13^C-metabolic flux analysis.

**Conclusion:**

Copper and its pivotal role to sustain cellular functions is noteworthy. However, knowledge on its cellular accumulation, availability and distribution for recombinant protein production is limited. This study attempts to address one such challenge, which revealed the fact that intracellular copper accumulation influenced laccase production and should be considered for high protein expression of copper-dependent enzymes when using *P. pastoris*. The results are discussed in the context of *P. pastoris* as a general host for copper -dependent enzyme production.

**Electronic supplementary material:**

The online version of this article (doi:10.1186/s12896-016-0251-3) contains supplementary material, which is available to authorized users.

## Background

Copper is an essential element for cellular metabolism. As part of prosthetic groups, it propels redox reactions for various copper-dependent enzymes including such as super oxide dismutase, cytochrome c oxidase, and the biocatalytically important laccase, tyrosinase [1], and amine oxidase [2]. Maintenance of copper homeostasis is a routine task for cells, and an imbalance needs to be met at the molecular level. In *Saccharomyces cerevisiae*, copper trafficking is accomplished through copper homeostasis factors [3]. At high copper concentrations, metallothionein-like proteins bind copper tightly and reactive oxygen species (ROS) are generated, thereby triggering a series of cellular damages such as lipid peroxidation, protein oxidation, and DNA damage [4–8]. At low copper concentrations, a hierarchy in copper distribution exists with mitochondria mostly preferred, followed by cytoplasm, and the Golgi apparatus. Transport of copper ions from the medium to the cells is initiated by membrane bound copper transporters like Ctr 1 and Ctr 3. These copper ions inside the cell are bound by copper homeostasis protein Atx1p and transported to the post-Golgi compartment of the secretory pathway. Here, Atx1p interacts with copper transporting P-type ATPase, located in the membrane of Golgi compartment. Ccc2p propels the copper ions delivered by Atx1p in to the lumen of the Golgi where copper is inserted in to the secreted copper-dependent enzymes [9–12].

Although the optimization of the production of various industrially relevant copper-dependent enzymes in yeast requires a quantitative description of the trade-off of copper availability and growth inhibition through copper to understand yeast physiology and morphology based on intracellular copper accumulation, only few studies have investigated this interplay. Growth is affected in different ways depending on the copper concentration. At low concentrations, it leads to cell morphogenesis, whereas at high concentration, it leads to stress response within the cells. Heavy metals were reported earlier to affect fruiting body, length of mycelium in fungi [13] or inhibition of mycelia development in *Candida albicans* [14]. Cellular adaptation of *Pichia guilliermondii* to high copper concentration was described by relating metabolic yield to growth rate. Growth of non-adapted cells were decreased with increasing copper concentration owing to high energy spent by the cells to protect from toxicity. Adapting the cells to high copper loads maintained constant growth rate, biomass yield and metabolic flux [15]. Through evolutionary engineering, *Candida* and *Saccharomyces* cells were able to acquire high copper tolerance. Changes in the level of detoxifying enzymes such as superoxide dismutase and catalase, regulation of copper uptake and alterations in the copper binding proteome conferred metal tolerance to the organism . Although there are no reports on copper uptake by *P. pastoris*, its peers such as *Rhodotorula spp* [16], *Candida utilis* [16] and *S. cerevisiae* [17] have been investigated for their ability to uptake copper.

Based on the Langmuir and Freundlich adsorption isotherms, cell metal binding sites, metal cation, ligand electronegativity, cavity size, and amount of copper adsorbed to the cell wall of yeast cells were measured [16]. The adsorbed copper fraction was deducted from the total copper for estimation of accurate intracellular copper concentrations.

A number of copper-dependent proteins are reported to be recombinantly produced using *P. pastoris*. In earlier reports, the copper concentration was varied to exclude copper limitation and hence to optimize recombinant protein production. Reported values range from 0.01 mM to 1 mM copper. Superoxide dismutase activity was enhanced upon 1 mM copper addition [18]; 0.05 mM to 0.5 mM copper was added to enhance amine oxidase expression [2]; copper levels up to 2 mM were shown to enhance tyrosinase expression from 2.5 mg/L to 24 mg/L [1] and hexose oxidase expression to 250 mg/L upon 10 μM copper addition to the medium [19]. Production of *T. versicolor* laccase and *Gaeumannomyces graminis* laccase in shake flask culture of *P. pastoris* was found to be 0.014 U/mL and 0.1 U/mL, respectively upon 0.4 mM copper supplementation to the medium [20]. Optimization of copper concentration to 0.4 mM improved laccase production in *P. pastoris* to 7.2 U/mL. Although the effect of copper on laccase production was reported at the level of transcription in some fungi, no such effect is expected in *P. pastoris* as laccase is under the control of the alcohol oxidase (AOX) promoter. Nevertheless, addition of copper sulphate to the medium most likely allows for correct folding of laccase in culture supernatant [21]. Copper and other metals such as magnesium, zinc, iron influence cell mass yields for either glucose- or methanol-grown *P. pastoris* cultures. Although added in trace amounts (as PTM1 metal stock solution) during cultivation in basal salts medium (BSM) [22], copper reserves within the cell need to replenished through regular supplementation for enhanced production of copper-dependent enzymes. Involvement of copper in the yeast metabolic pathway is notable. Especially, its share as a cofactor for functional activation of several enzymes involved in copper metabolism (23 %), respiration (15 %), TCA cycle (9 %) and amino acid metabolism (8 %) is significant [23]. Copper deficiency may cause the enzymes aconitase and succinate dehydrogenase of the TCA cycle to become rate-limiting upon diauxic shift and affect the biosynthetic pathways for lysine, arginine, aromatic amino acids, cysteine, and methionine [24]. Hence, the metal concentration in the medium for *P. pastoris* plays a decisive role in recombinant protein production. Notably, the effect of changing the copper concentration in the medium was found in earlier reports to not be correlated to the copper uptake by *P. pastoris*. Hence, a better insight into copper accumulation and its impact on protein production could not be derived.

The stringent requirement to maintain cellular copper levels within the limit of adequacy to meet metabolic demand, and below those that exceed the capacity of the cell to appropriately bind and store copper to prevent cytotoxicity, demands a sensitive attentiveness towards dynamic fluctuations in extracellular and intracellular copper. Although natural hosts produce active laccase, heterologous hosts can produce engineered laccase or other copper-dependent enzymes with desirable properties, such as multiple substrate specificity, stability for various industrial and biotechnological applications. Yeasts, in particular, *P. pastoris* attracts an excellent market, for large scale production of copper containing enzymes through its innate ablity of copper chaperones to acquire and distribute copper ions, while maintaining non-toxic conditions. Thus, in this study, the impact of beneficial and detrimental copper concentrations on the growth of *P. pastoris,* enhancement of laccase expression through copper and the improvement of specific growth rate through laccase expression were studied. The intracellular copper concentration was estimated with the goal of reporting a strategy to determine the optimum copper concentration for enhanced laccase production that is extendable to other copper-dependent proteins produced with *P. pastoris.*^13^C metabolic flux analysis was conducted to ascertain the flux redistribution through central carbon metabolism. The reported trade-off between copper availability and growth inhibition allows for improved copper-dependent recombinant protein production in *P. pastoris*.

## Results and Discussion

### Effect of copper load on the growth of *P. pastoris*

**Fig. 1:**
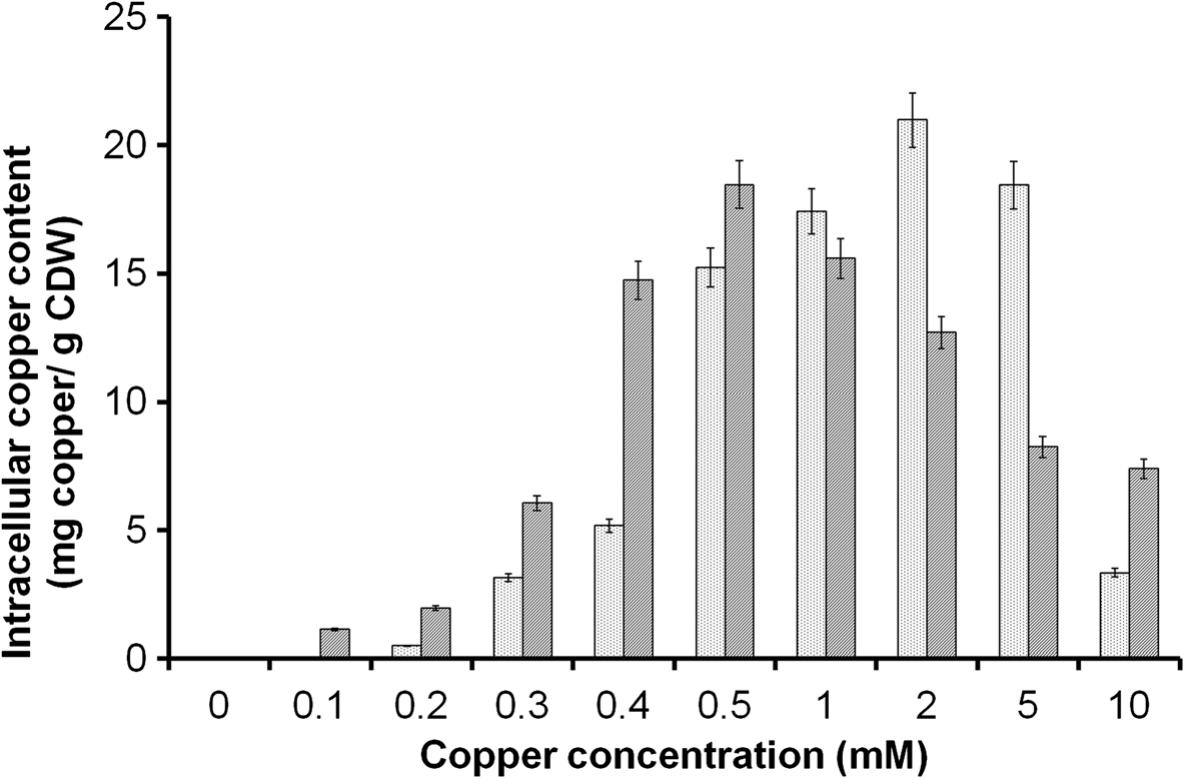
Intracellular specific copper content of *P. pastoris* wildtype () and GSLAC () cultivated in the presence of various copper concentrations. Cells were harvested during exponential growth phase. Intracellular copper accumulation is reported excluding the adsorbed copper. The values are arithmetic mean of two biological experiments. Error bars indicate deviation from the mean

**Fig. 2:**
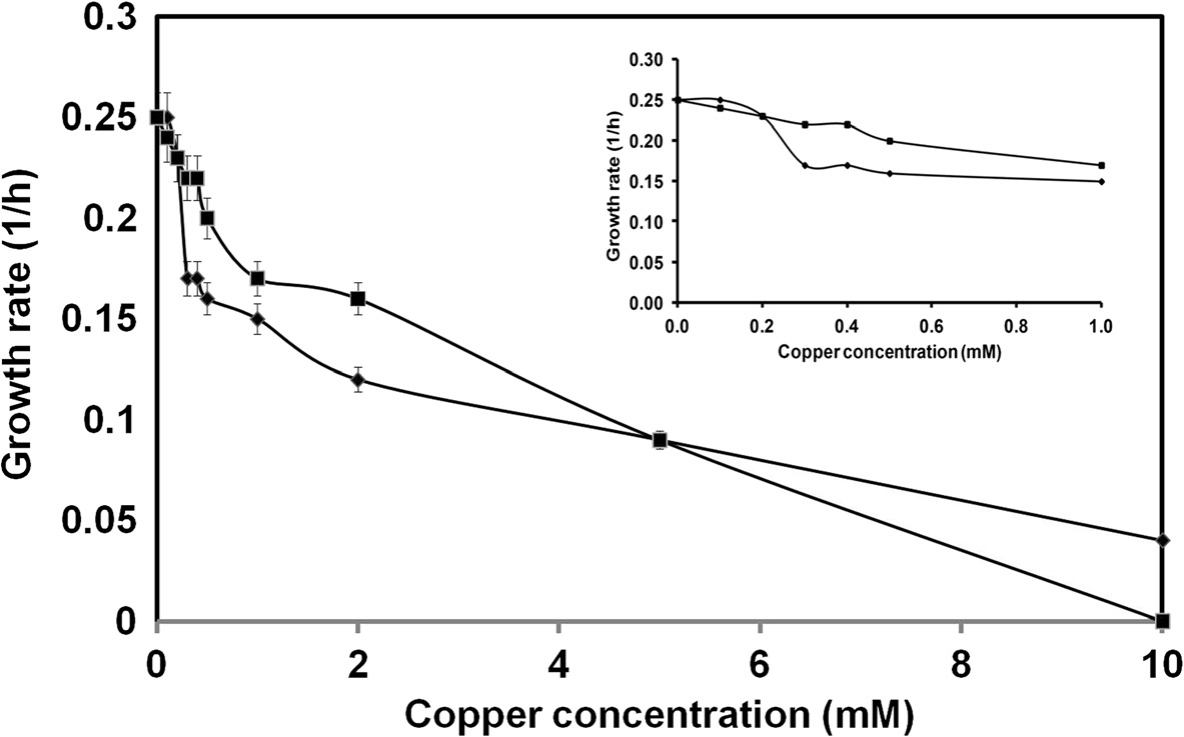
Effect of increasing copper concentration on the growth rate of *P. pastoris*. Growth was monitored every 3 h during cultivation in BMDH medium. The symbol  indicates wildtype and  indicates GSLAC. Concentration from 0.1 mM to 1 mM was zoomed and shown as a smaller graph. The values are arithmetic mean of two biological experiments. Error bars indicate deviation from the mean

**Fig. 3:**
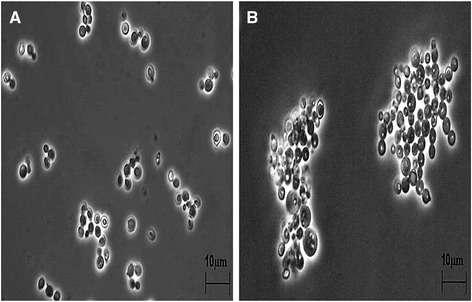
Influence of copper concentration on the morphology of *P. pastoris*. **a** Control (without added copper) and (**b**) cells grown in the presence of 2 mM copper. BMDH was used as growth medium. Pictures were taken using a Leica ICC50 microscope with 100x magnification

Varying copper concentrations ranging from 0.1 mM (6.35 mg/L) to 50 mM (3,175 mg/L) were used for copper-based measurements in GS115 laccase (GSLAC) and wild type strains. These wide ranges of concentrations were chosen to determine the beneficial and adverse effects of copper on *P. pastoris*. Absence of added copper did not result in copper-free medium as medium constituents like yeast nitrogen base (YNB) carry trace amounts of copper (0.04 mg/L, (Sigma, USA)). The detection limit of the used atomic absorption spectrophotometer (AAS) was 1 mg/L, indicating that the residual copper concentration in the 0 mM sample was below this value (Fig. 1). Notably, growth was already slightly affected at low concentrations of additional copper, whereas 1 mM of copper (μ = 0.15 h^−1^) reduced the growth rate by 40 %. Increasing copper concentrations from 2 mM (127 mg/L) to 10 mM (635 mg/L) reduced the growth rate significantly from 0.12 h^−1^ to 0.04 h^−1^. High intracellular copper accumulation beyond the threshold level (21 mg_copper_/g_CDW_) appeared to commence cellular toxicity leading to a decline in biomass and hence a decreasing copper content (Additional files 1-2). In addition, the lag phase became more pronounced for higher copper loads. For copper concentrations above 10 mM, no growth was observed (Fig. 2). Intracellular copper concentrations increased from 0.04 mg_copper_/g_CDW_ to 21 mg_copper_/g_CDW_ at 0.1 and 2 mM extracellular copper, respectively. The obtained values correlated with values from *P. guillermondii* (20 mg_copper_/g_CDW_) [15], *Rhodotorula spp* (16.8 mg_copper_/g_CDW_) [16], *C. utilis* (16.2 mg_copper_/g_CDW_) [16], and *S. cerevisiae* (12.6 mg_copper_/g_CDW_) [17]. In *S. cerevisiae* and 16 additional yeasts, copper adsorption was higher at low biomass concentration, and the copper accumulated in biomass increased with increasing concentrations of copper [16]. The results expound the interaction between intracellular copper accumulation and growth rate. Presently, modelling and model-based feeding control strategies are available [25–29] to improve the growth rate in *P. pastoris.* Cell agglomerates occurred at high copper concentrations (Fig. 3). Electrostatic interaction between positively charged metal ions and the negatively charged cell wall, which leads to charge neutralization, might allow the neighbouring cells to aggregate and not repel [30] and could be one reason for agglomerates.

### Laccase expression attenuates the impact of copper on specific growth rate of *P. pastoris*

A decrease in the growth rate of *P. pastoris* with ascending copper concentrations led to a search for the constraint as it is necessary to maintain copper homeostasis without compromise on growth rate for enhanced recombinant protein production in *P. pastoris*. Grouped in the family of oxidoreductases, laccases have been shown to be truly multifunctional enzymes. Earlier studies on functional expression of laccase in *P. pastoris* reported an enhanced resistance to H_2_O_2_ [31] or resistance to paraquat and menadione induced oxidative stress in *P. pastoris* strain overexpressing Cu - Zn Superoxide dismutase [32]. Figure 2 compares the growth rates of GSLAC with *P. pastoris* wild type under various copper concentrations. Notably, laccase expression (3 U/mL) correlated with a higher growth rate, which decreased from 0.25 h^−1^ to 0.22 h^−1^ when copper concentration was increased from 0.1 mM (6.35 mg/L) to 0.5 mM (31.75 mg/L), respectively. Under these conditions, the growth rate of *P. pastoris* wild type declined to 0.15 h^−1^, indicating a beneficial role for laccase expression on growth of *P. pastoris* during copper exposure. A similar effect was observed with *S. cerevisiae* where laccase expression under gal1 promoter control maintained normal growth, whereas chitinase led to growth inhibition [33]. Furthermore, laccase triggers genes involved in the glutathione-based antioxidative system, thereby conferring stress protection and growth maintenance in *P. pastoris*. Thus, copper-dependent enzymes could be considered as an alternative to sustain the growth rate in *P. pastoris*.

### Intracellular copper levels influence laccase production

**Fig. 4:**
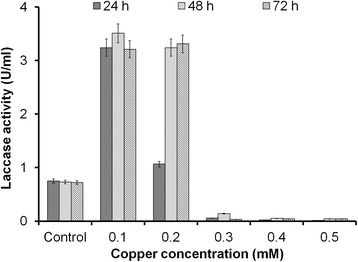
Effect of copper concentrations (0.1 mM to 0.5 mM) on laccase activity. BMDH medium (pH 5.0) was used for growth of *P. pastoris*. Laccase activity in the supernatant was quantified using the DMPPDA assay. The values are arithmetic mean of two biological experiments. Error bars indicate deviation from the mean

**Fig. 5:**
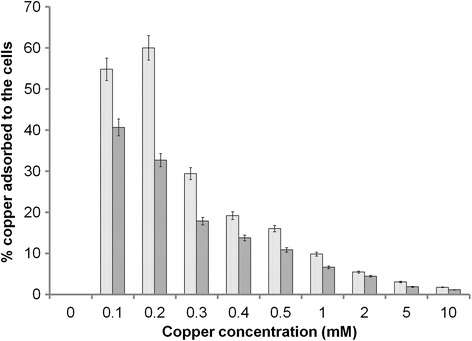
Fraction of copper adsorbed to *P. pastoris* wildtype () and GSLAC (). Inactivated cells were incubated in the presence of different copper concentrations for 30 min. Cells were then analyzed for their copper content to estimate copper adsorption of the cell wall. The values are arithmetic mean of two biological experiments. Error bars indicate deviation from the mean

**Fig. 6:**
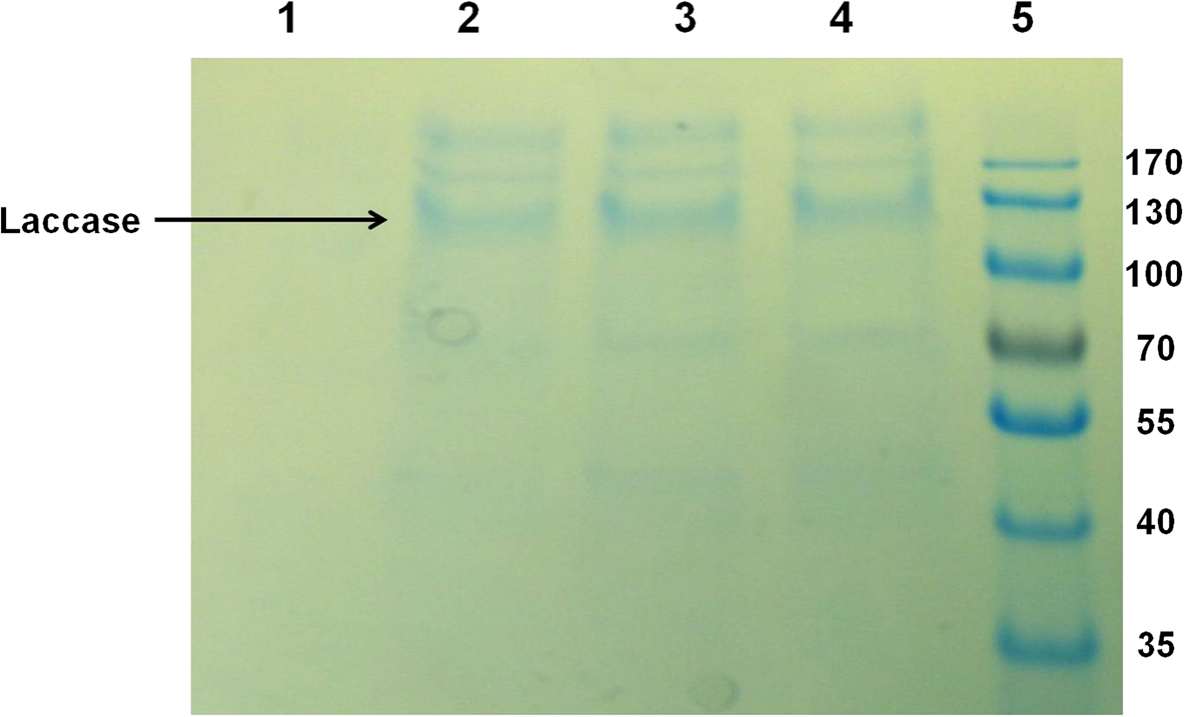
SDS PAGE analysis of laccase expressed by GSLAC at 0.1 mM copper concentration. Lane1: Sample collected at 0 h; Lane 2 : Sample collected at 24 h; Lane 3 : Sample collected at 48 h; Lane 4 : Sample collected at 72 h; Lane 5: Molecular weight marker. 13 μg of protein was loaded in each lane

Copper as an essential cofactor has to be sufficiently provided for augmented laccase production. Although no systematic analysis on the influence of copper concentration on recombinant protein production exists, reported values are in the range of 0.1 mM to 0.5 mM copper [34–53]. In this study, a fourfold higher laccase activity (3 U/mL) of GSLAC (under the control of GAP promoter) was observed with 0.1 mM and 0.2 mM copper in the medium (Fig. 4) compared to the control (0.75 U/mL). An expression study was conducted in a shake flask with buffered minimal dextrose medium with histidine (BMDH) medium, and activity was checked at 24, 48 and 72 h. Conversely, 0.3 mM (19.05 mg/L) to 0.5 mM (31.75 mg/L) of copper in the medium correlated with significantly lower laccase production (0.05 U/mL to 0.14 U/mL). Traditionally, the total copper accumulated by yeast is determined by modifications of the earlier method [54]. Copper accumulation by cells is estimated using adsorption models [55] or physical/chemical disruption techniques followed by inductively coupled plasma mass spectrometry (ICP-MS) or AAS analysis [56, 57]. Notably, copper adsorption to the cell wall is rarely reported, although yeast are used as unspecific metal adsorbers [58]. Here, we determined the actual intracellular copper concentration by subtracting the adsorbed copper amount from the total. There was 40 % copper adsorption at 0.1 mM, which reduced further to 4 % at 2 mM. This result clearly showed that the copper adsorbed to the cells is dependent on the biomass concentration (Fig. 5). Although *P. pastoris* was found to accumulate a high copper load in the range of 6 to 18.5 mg_copper_/g_CDW_, intracellular copper content within the span of 1.1 to 1.9 mg_copper_/g_CDW_ (at 0.1 mM copper) was found to enhance laccase expression (Fig. 1). One explanation for this counterintuitive result is that excess copper binds unspecifically to copper-independent proteins, thereby causing loss of function including protein denaturation [59]. It was reported that copper may disrupt the three dimensional structure of proteins in the secretory pathway by binding to sulphur or carboxyl containing groups, thereby disrupting vital biochemical reactions [60]. Shake flask studies with 0.1 mM copper concentration showed that the level of laccase expression at different time periods of 0, 24, 48 and 72 h remained the same (Fig. 6).

### Unchanged flux distribution through central carbon metabolism under varying copper concentrations

To verify whether there was a competitive regulation of copper through the metabolic network apart from copper trafficking pathway, the physiology of laccase expressing *P. pastoris* (GSLAC) was quantified by ^13^C metabolic flux analysis. The physiology of a GSLAC strain expressing laccase under the control of constitutive GAP promoter (with 0.1 mM and 0.3 mM copper and without copper) and a wild type strain (with and without copper) was analysed. Both wild type and the clone showed no significant changes with or without copper supplementation and hence the data obtained for wildtype without copper and the GSLAC clone with 0.1 mM and 0.3 mM were chosen for comparison. Except for the distribution of duplicate genes, the central carbon metabolism of *P. pastoris* and *S. cerevisiae* are identical [61–64]. Hence, a metabolic model of *S. cerevisiae* was used for flux calculation (Additional files 3-9).

**Fig. 7:**
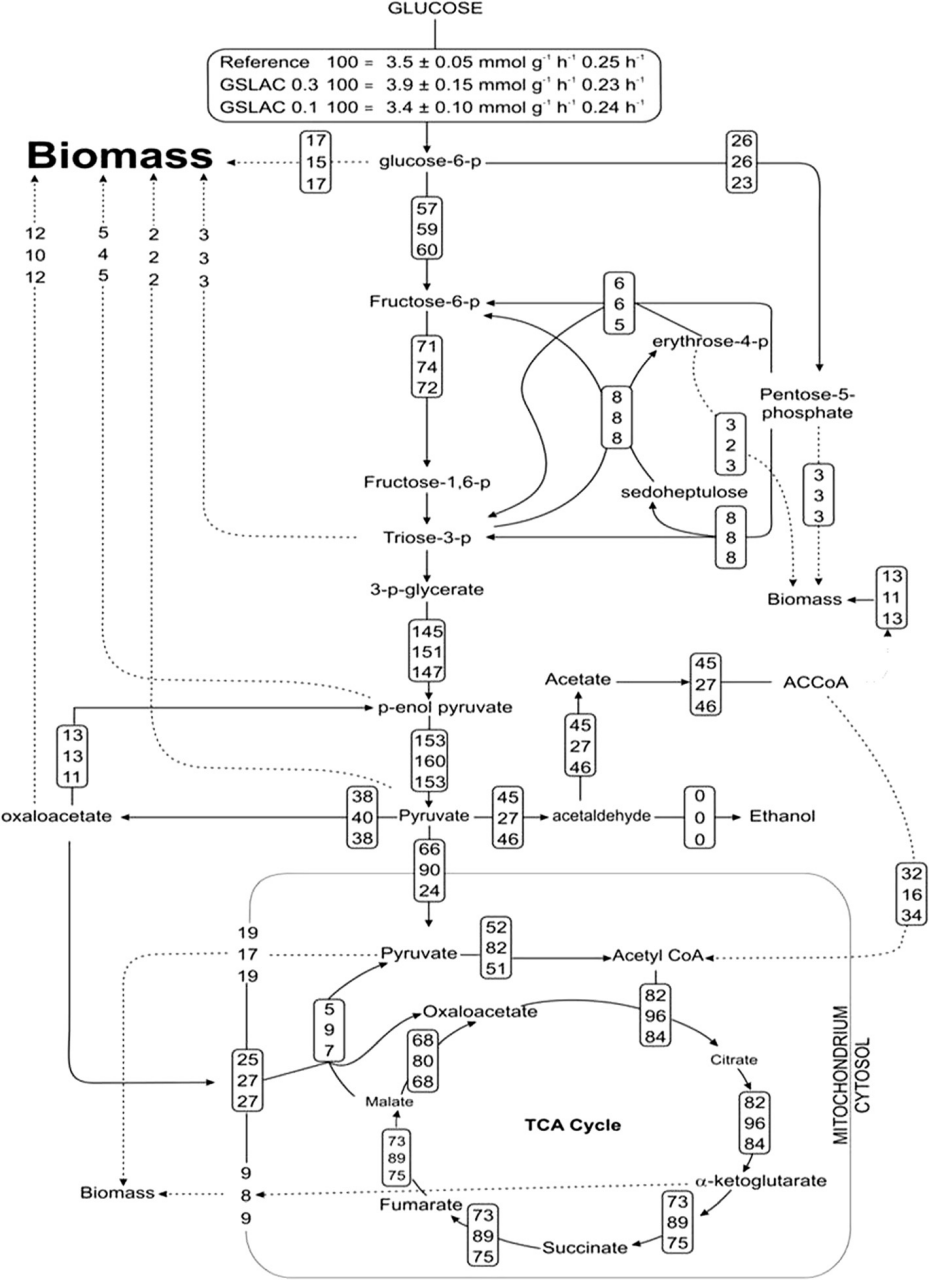
Metabolic flux distribution in *P. pastoris*. Distribution of relative carbon fluxes in the *P. pastoris* wildtype strain (top) and laccase producing GSLAC strain at 0.3 mM (middle) and 0.1 mM copper (bottom) during exponential growth in batch cultures. Fluxes were normalized to specifc glucose uptake rate shown in the top box

Shake flask cultivations of wildtype and recombinant strains were performed under pseudo-steady state conditions. Laccase titres of approximately 0.4 U/mL (at 0.1 mM and 0.2 mM copper) were attained at a biomass concentration of 2.4 g_CDW_/L. Both the reference and the recombinant strain had very low pyruvate and fumarate production rates of 0.06 mmol g^−1^ h^−1^ and 0.006 mmol g^−1^ h^−1^, respectively. Acetate and ethanol were not detectable in the supernatant. Despite a slight difference in growth rate, the glucose uptake rate was in the range of 3.7 to 4.2 mmol g^−1^ h^−1^ for the wild type and GSLAC strain. This comparable glucose influx was highly similar distributed in the metabolic network (Fig. 7); hence, the copper concentrations favourable for laccase production did not cause a change in central carbon metabolism operation. Similar results were obtained with *P. guilliermondii* [15], whereas a transient shift to the pentose phosphate pathway, to sustain NADPH regeneration to counter oxidative stress, was induced by high copper loads [65].

A single *Pichia* cell with a 5 μm diameter and a volume of 65 femtolitre (fL) [66] produced nearly 3.03 x 10^−12^ micromoles of laccase. Hence, 12.12 x 10^−12^ micromoles of copper per cell are required (1 mole of laccase requires 4 moles of copper) for functional laccase expression. Remarkably, a single *P. pastoris* cell accumulated 0.12 x 10^−9^ micromoles of copper at 0.1 mM external copper, or an about 10-fold copper excess. Most likely, the copper did not reside in only the cytosol and hence was accessible for laccase production but was stored at high amounts in the vacuoles [67]. This possibility of *P. pastoris* to maintain non-toxic conditions allows the production of laccase (Table 1) and other copper dependent enzymes from *P. pastoris* at high rates.

**Table 1:** Expression of different laccases in *P.* pastoris

Laccase source	Laccase activity (U/mL)	Promoter	Reference
*Aspergillus clavatus* ^a^	3	GAP	This work
*Bacillus amyloliquifaciens* ^c^	0.4	AOX1	[72]
*Botrytis aclada* ^a^	51	AOX1	[34]
*Cerena spp.* ^b^	6	AOX1	[73]
*Ganoderma fomicatum* ^b^	4	AOX1	[74]
*Ganoderma Lucidum* ^b^	0.7	AOX1	[46]
*Pleurotus sajor-caju* ^b^	10	AOX1	[75]
*Polyporus grammocephalus TR16* ^b^	0.3	AOX1	[45]
*Rigidosporus microsporus* ^b^	9	GAP	[76]
*Thermus thermophilus SG0.5JP17-16*	6	AOX1	[77]
*Trametes spp. laccase B* ^b^	32	AOX1	[78]
*Trametes versicolor* ^b^	0.003	GAP	[79]
*Trametes spp. AH28-2* ^b^	5	AOX1	[80]
*Trametes versicolor lccB* ^b^	33	AOX1	[81]
*Trametes trogii* ^b^	3	AOX1	[82]

^a^ Ascomycete; ^b^ Basidiomycete; ^c^ Bacteria

## Conclusion

Copper as an essential metal has to be provided in sufficient amounts within the medium to allow growth at a maximal rate and, therefore, to yield high biomass and protein formation by *P. pastoris*. The latter is of interest for recombinant protein production, especially of copper-dependent proteins such as laccases. In this work, a simple method to determine the intracellular copper concentration is presented. Although *P. pastoris* accumulates copper at high intracellular concentrations, 1.1 to 1.9 mg_copper_/g_CDW_ was sufficient for high recombinant laccase expression. This range of intracellular copper concentration allows high growth rates. Further increase of the copper content in the growth media decreases the growth rate and recombinant protein production drastically. The obtained knowledge is very likely transferable to other copper-dependent proteins that can be produced with *P. pastoris*.

## Methods

### Strains and culture conditions

*Pichia pastoris* GS115 (*his4*) (wild type) purchased from Invitrogen was used throughout the study. YPD (1 % (w/v) yeast extract, 2 % (w/v) peptone, 2 % (w/v) dextrose) medium was used for the pre-culture (500 mL flask with 50 mL medium, 200 rpm, an amplitude of 25 mm and 30 °C). Buffered minimal dextrose medium with histidine (BMDH) (2 % (w/v) dextrose, 100 mM potassium phthalate buffer pH 5.0; 1.34 % (w/v) YNB; 4*10^−5^% (w/v) biotin) was used for copper analysis and tolerance studies. The laccase gene [EMBL: EAW07265.1] from *Aspergillus clavatus* was codon optimized by Gen Script Corp (Piscataway, NJ). The pGAPZαA plasmid was purchased from Invitrogen to construct the pGAPZαA laccase construct. *P. pastoris* GS115 expressing laccase (GSLAC) was constructed according to the easy select Invitrogen protocol (EasySelect^TM^*Pichia* expression kit) and used in this study for comparison with the empty *P. pastoris* GS115 strain. Copper sulphate pentahydrate (CuSO_4_·5H_2_O) was used throughout the study as a copper source. All reagents and chemicals used in this study were of analytical grade.

### Determination of cellular copper

During cellular copper determination and adsorption studies, the strains (GSLAC and wildtype) were inoculated at 0.5 OD_600nm_ (0.13 g_CDW_/L) and grown in BMDH medium up to exponential phase in shake flasks. Cells harvested in exponential phase were centrifuged at 2968 x g for 10 min at room temperature. The supernatant was collected and stored at 4 °C for extracellular copper measurement. The biomass was washed twice with distilled water (to remove the media components and impurities) and finally suspended in 10 mL of distilled water. Then, 1 mL of the biomass was withdrawn for cell dry weight measurement, whereas the remaining 9 mL biomass was centrifuged again at 2968 x g for 10 min. This remaining biomass was suspended in 9 mL of 6 M nitric acid (HNO_3_) overnight for complete cell lysis. The cell lysate contains both intracellular copper and those copper adsorbed to the cell wall. Hence, the actual intracellular copper present is determined by deducting the copper adsorbed to the cell wall. A Perkin Elmer (Perkin Elmer, Waltham, MA, USA) 1100b atomic absorption spectrophotometer with an air-acetylene flame (acetylene 2.6 from Westfalen AG, Münster, Germany; Airflow: 8 L/min; acetylene flow: 2.5 L/min) was used for determining the copper content of cells. A copper hollow lamp was operated at 324.7 nm with a spectral bandwidth of 1.0 nm and a lamp current of 10 mA. Analyses were performed using peak height mode to determine absorbance values. All experiments were conducted in duplicate. For copper adsorption, cells were harvested by centrifugation at 2968 x g for 10 min. The harvested cells were suspended in 25 mL double distilled water, mixed well and heated in a water bath (to inactivate the cells) maintained at 55 °C for 30 min. Cell pellets were resuspended in 50 mL of BMDH medium containing varying copper concentrations and these cell slurries were incubated for 2 h at room temperature. The obtained supernatant was used for extracellular copper analysis. The harvested cell pellet was subjected to cell lysis as described above [68].

### Laccase assay

*N*,*N*-dimethyl-*p*-phenylenediamine (DMPPDA) was used as substrate for the laccase. The assay was carried out in microwell plates with a total reaction volume of 200 μL. The reaction mixture contained 50 μL of culture supernatant, 50 mM sodium acetate buffer (pH 5.0), and 5 mM DMPPDA. The colour change was monitored continuously for 10 min at 550 nm and 30 °C [69].

### Analysis of laccase expression

Sodium dodecyl sulphate polyacrylamide gel electrophoresis (SDS-PAGE) was performed on culture supernatants using Any kD^TM^ precast protein gels (Biorad, USA). The supernatants of strain GSLAC was heat denatured by heating at 100 °C for 10 min in denaturing buffer containing SDS and 2-mercaptoethanol. The proteins were stained with Coomassie Brilliant Blue R-250 (Carl Roth GmbH, Germany). The amount of laccase in supernatants expressed by GSLAC was quantified using bovine serum albumin (Carl Roth GmbH, Germany) as standard.

### Growth physiology analytics

Exponentially growing cultures were used as pre-inoculum for starting the growth experiments. When an OD_600nm_ of 0.5 was reached, the biomass concentration of *P. pastoris* was continuously monitored every 3 h up to exponential phase and harvested. Pseudo-steady state was achieved during the exponential growth phase during which the physiology of the cell population remained constant. To verify pseudo-steady state conditions, extracellular rates such as substrate uptake rate, growth rate, product formation rate, metabolite production rate were determined. Extracellular metabolites were analysed through HPLC. Analytes were separated using an organic acid resin column (CS Chromatographie Service GmbH, Langerwehe, Germany) at 50 °C. 5 mM sulphuric acid was used as eluent at a flow rate of 0.8 mL min^−1^ (System Gold 125 Solvent Module, Beckman Coulter, USA). Analytes were detected with a UV detector (System Gold 166 UV-Detector, Beckman Coulter, USA) at a wavelength of 210 nm and a RI detector (Melz Differential Refractometer LDC 201, Germany) operated at 25 °C.

For metabolic flux analysis, samples of 0.3 mg CDW each were taken during exponential growth for the determination of ^13^C incorporation into proteinogenic amino acids. After centrifugation (2968 x g, 4 °C), the supernatant was discarded and the pellet washed with deionized water and centrifuged again. The cell pellet was resuspended in 150 μL 6 M HCl and hydrolysed at 105 °C for 6 h. The cell hydrolysates were dried at 85 °C under the hood, and the water-free sample was resuspended in 30 μL acetonitrile and derivatized with 30 μL of N-methyl-N-(tert-butyldimethylsilyl) trifluoroacetamide (MBDSTFA) for one hour at 85 °C and analysed by GC-MS directly afterwards.

Gas chromatography was carried out with a Thermo Scientific (ThermoScientific, Waltham, MA, USA) Trace GC Ultra equipped with a ThermoScientific TriPlus RSH™ Autosampler. Fifteen detectable amino acids were separated on a Restek Rxi-5Sil MS (Length: 15 m; Inner diameter: 0.25 mm; Film: 0.25 μm) column at a constant flow rate of 1 mL helium min^−1^ (Helium 5.0 from Praxair Deutschland GmbH, Düsseldorf, Germany). A sample volume of 1 μL was injected into a PTV injector in a constant temperature injection at 270 °C while a split ratio of 1:40 was used. The temperature of the GC oven was kept constant for 1 min at 140 °C and afterwards increased to 270 °C with a gradient of 10 °C min^−1^.

Mass spectrometry analysis was performed on a ThermoScientific (ThermoScientific, Waltham, MA, USA) TSQ triple quadrupole mass spectrometer. The temperatures of the transfer line and the ion source were both set to 280 °C. Ionization was performed by electron impact (EI) ionization at 70 eV and all masses between 180 and 550 m/z were detected in a scantime of 0.25 s. GC-MS raw data were analysed using the software Xcalibur.

### ^13^C metabolic flux analysis

Cell growth was monitored continuously by measuring the optical density at 600 nm (OD_600_). Cells were grown in BMDH medium containing 5 g/L glucose. Cells were harvested during mid-exponential phase of 13 h to 16 h (2 – 4 OD_600_). All the experiments were conducted in duplicates. The glucose used in the ^13^C tracer experiment was a mixture of 20 % (n/n) uniformly labelled [U-^13^C] glucose (99 %, Cambridge Isotope Laboratories, USA) and 80 % (n/n) naturally labelled glucose. The metabolic model comprised major pathways of central carbon metabolism of yeast. Four extracellular flux parameters (growth rate, production rate of pyruvate and fumarate, glucose uptake rate) and five intracellular flux parameters (fraction of cytosolic oxaloacetate originating from cytosolic pyruvate, mitochondrial oxaloacetate derived through anaplerosis, fraction of phosphoenol pyruvate originating from cytosolic oxaloacetate, and fraction of serine derived through glycolysis) were used [25]. Fluxes were estimated using FiatFlux [70]. Metabolic flux model was equipped with published biomass composition of *P. pastoris* [71].

## Additional files

Additional file 1:
**Intracellular copper content and copper adsorption.** (XLS 26 kb) Additional file 2:
**Percentage copper adsorbed to the cells.** (XLS 19 kb) Additional file 3:
**Extracellular flux parameters data for GSLAC clone**. XLS 38 kb) Additional file 4:
**Extracellular flux parameters data for wildtype.** (XLS 38 kb) Additional file 5:
**Flux ratios and mass distribution vectors of amino acids.** (XLS 192 kb) Additional file 6:
**Carbon source and Optical density of shake flask cultivation. **(XLS 20 kb) Additional file 7:
**SDS PAGE analysis of wildtype.** (XLS 251 kb) Additional file 8:
**Net fluxes of wildtype.** (XLS 81 kb) Additional file 9:
**Net fluxes of GSLAC clone.** (XLS 81 kb)

## Abbreviations

AAS: Atomic absorption spectrophotometer
AOX: Alcohol oxidase
BMDH: Buffered minimal dextrose medium with histidine
BSM: Basal salts medium
CuSO_4_· 5H_2_O: Copper sulphate pentahydrate
DMPPDA: *N*,*N*-Dimethyl-*p*-phenylenediamine
GAP: Glyceraldehyde - 3 - phosphate dehydrogenase
GSLAC: GS115 laccase
ICP-MS: Inductively coupled plasma emission mass spectrometry
MBDSTFA: N-methyl-N-(tert-butyldimethylsilyl) trifluoroacetamide
ROS: Reactive oxygen species
YNB: Yeast nitrogen base
